# Integrating occurrence and detectability patterns based on interview data: a case study for threatened mammals in Equatorial Guinea

**DOI:** 10.1038/srep33838

**Published:** 2016-09-26

**Authors:** Chele Martínez-Martí, María V. Jiménez-Franco, J. Andrew Royle, José A. Palazón, José F. Calvo

**Affiliations:** 1 Departamento de Ecología e Hidrología, Facultad de Biología, Universidad de Murcia, Campus de Espinardo, 30100 Murcia, Spain; 2 USGS Patuxent Wildlife Research Center, 12100, Beech Forest Road, Laurel, MD 20708, USA

## Abstract

Occurrence models that account for imperfect detection of species are increasingly used for estimating geographical range, for determining species-landscape relations and to prioritize conservation actions worldwide. In 2010, we conducted a large-scale survey in Río Muni, the mainland territory of Equatorial Guinea, which aimed to estimate the probabilities of occurrence and detection of threatened mammals based on environmental covariates, and to identify priority areas for conservation. Interviews with hunters were designed to record presence/absence data of seven species (golden cat, leopard, forest elephant, forest buffalo, western gorilla, chimpanzee and mandrill) in 225 sites throughout the region. We fitted single season occupancy models and recently developed models which also include false positive errors (i.e. species detected in places where it actually does not occur), which should provide more accurate estimates for most species, which are susceptible to mis-identification. Golden cat and leopard had the lowest occurrence rates in the region, whereas primates had the highest rates. All species, except gorilla, were affected negatively by human settlements. The southern half of Río Muni showed the highest occurrence of the species studied, and conservation strategies for ensuring the persistence of threatened mammals should be focused on this area.

The determination of species occurrence through site occupancy models in order to investigate species distribution, status and threats is a fundamental step in the management and conservation of endangered species^1^,^2^. As a consequence of rapid human population growth and agriculture development, most large mammal species are threatened globally by overhunting and habitat fragmentation, leading to a loss of biodiversity and species extinctions, especially in Africa and south-east Asia^3^,^4^. Potential factors of occupancy can be modelled as a function of covariate information^5^, such as landscape characteristics or human densities^6^,^7^, enabling researchers to focus on different aspects of conservation and at different scales, ranging from global^8^ or regional^6^ to local^9^,^10^,^11^.

Río Muni, the continental region of Equatorial Guinea (Central Africa), is one of the most diverse regions for mammals and contains priority areas for global mammal species conservation^8^. This area has among the highest concentrations of threatened terrestrial mammals in the world because of habitat loss, so the country is promising for targeting large mammal conservation efforts. This is particularly true in the densely populated Biafran region as: (1) the movement of rural people to urban centres given impetus by the oil and gas sectors^12^ generates opportunities for the creation of new conservation areas, and (2) existing legislation protects several species and prohibits hunting within protected areas. However, there is basically no effective on-the-ground protection, and evidence of human hunting inside protected areas are found^13^.

The aims of this study were to estimate the probability of occurrence and detection of threatened mammals in relation to environmental and human disturbance covariates through a regional-scale case study in Río Muni and to develop corresponding species-specific distribution maps which may help to identify priority conservation areas. For this, we selected seven mammal species of conservation interest: golden cat (*Caracal aurata*), leopard (*Panthera pardus*), forest elephant (*Loxodonta cyclotis*), forest buffalo (*Syncerus caffer*), western gorilla (*Gorilla gorilla*), chimpanzee (*Pan troglodytes*) and mandrill (*Mandrillus sphinx*).

When large and unknown areas are difficult and costly to survey through classical survey techniques based on field observations, public sightings or well-designed interviews can provide a cost-effective alternative monitoring technique^14^. In recent years, initiatives to analyse large and extensive data sets collected by hunter interviews have increased^15^,^16^,^17^,^18^,^19^. However, this approach can present a challenge, as sympatric species that are morphologically similar, rare or elusive, can be particularly vulnerable to misidentification^20^.

Given the inconspicuous nature of most animals, occurrence surveys can be designed that allow for imperfect detection (e.g. false negatives, or failure to detect a species where in fact the species is present). In order to minimize the possibility of false absences or false negatives, multiple surveys of the sampling units within a relatively short timeframe are conducted^5^,^21^. Another problem associated with sampling and detection are false positives, i.e., a species detected where it does not occur^22^,^23^,^24^,^25^. Therefore, recent occupancy models have focused on the need to also take into account false positives errors, e.g., in surveys of many taxa involving the simultaneous sampling of large numbers of species by volunteer observers with variable skill levels^22^, in acoustic surveys^26^,^27^,^28^ and in multiple detection method models with certain and uncertain detections^29^,^30^.

We used single season occupancy models to analyse species presence/absence data collected from interviews with local wildlife hunters. Most occupancy models based on interview studies do not consider false positive estimates^16^,^17^, and assume that all reports of presence must represent certainty. However, recent occupancy studies based on hunter observation of wolves have taken into account false positive estimates^15^,^31^, suggesting that data from hunter interviews is uncertain and likely to include observation errors which may be due to observer inexperience and/or the sensitivity of hunters to recent controversy concerning the studied species. Just as in these studies, we also think that hunter wildlife knowledge can be reasonably questioned. Therefore, we considered both conventional models (which account for false negative errors) and misclassification models (which account for false negatives and false positives) to identify environmental and disturbance covariates which influence species occupancy and detectability. Our predictions about the influence of covariates on occurrence patterns were species-specific (Supplementary Table S1), but in general we expected negative relationships with human density and positive relationships with ruggedness and the extent of forest area within the hunting sites.

## Results

Our results show that models including ecological (elevation, ruggedness and forest area) and disturbance covariates (human population density) were strongly favoured for all studied species (Supplementary Table S2). The importance of the four covariates in parameter estimates was clear, although there was considerable variability regarding the best combination of these variables. For all species, except chimpanzee, the best model had a higher number of covariates, including interactions (more complex models). For example, the best model for forest buffalo included all the studied covariates both in the occurrence (ψ) and detection (*p*) probability estimates (Table 1). Moreover, misclassification models were the best explaining models for most of the studied species (leopard, forest elephant, western gorilla, chimpanzee and mandrill; Table 1).

The top ranked models (with ∆AIC < 2.0; see bold AIC values in Supplementary Table S2) for leopard, forest elephant, western gorilla and chimpanzee included only misclassification models, whereas for forest buffalo and mandrill included both conventional and misclassification models. Top ranked models for golden cat included non-misclassification models only. The highest estimated misclassification rate was 0.056 for chimpanzee and the lowest was 0.005 for mandrill (Table 1). Note that the false positive rate parameter (*p*_10_) is zero in non-misclassification models.

With regards to the effects of the covariates considered, our results support most of our predictions (Supplementary Table S1). Table 1 shows the untransformed coefficients of covariates of the top ranked models, which influence the probabilities of occurrence and detection of the studied mammals. Elevation was included in the top models for forest buffalo, with a negative effect on occupancy, a tendency that could be extended to forest elephant, western gorilla and mandrill. Ruggedness was included in all the top models for golden cat, leopard, forest buffalo, western gorilla and mandrill with a positive effect on occupancy. As we predicted, forested area had a positive effect on occupancy for forest elephant and forest buffalo, but was not relevant in the case of gorilla and chimpanzee. Contrary to our predictions, more highly forested areas was not found relevant for the occupancy of leopard and showed a negative effect in the case of golden cat. The density of human settlements had a negative effect on occupancy for all species except western gorilla.

Covariates which determine detection probabilities in the best models followed similar tendencies to covariates in occupancy models both in forest buffalo and mandrill (Table 1). Unlike covariates which influenced occupancy estimates from the top models, elevation was included in the best models for golden cat and leopard, with a positive effect on *p*. Forest area was in the top model of detection probability estimates for elephant, with a positive effect, and human population density had a negative effect on the detection probability of western gorilla.

**Figure 1:**
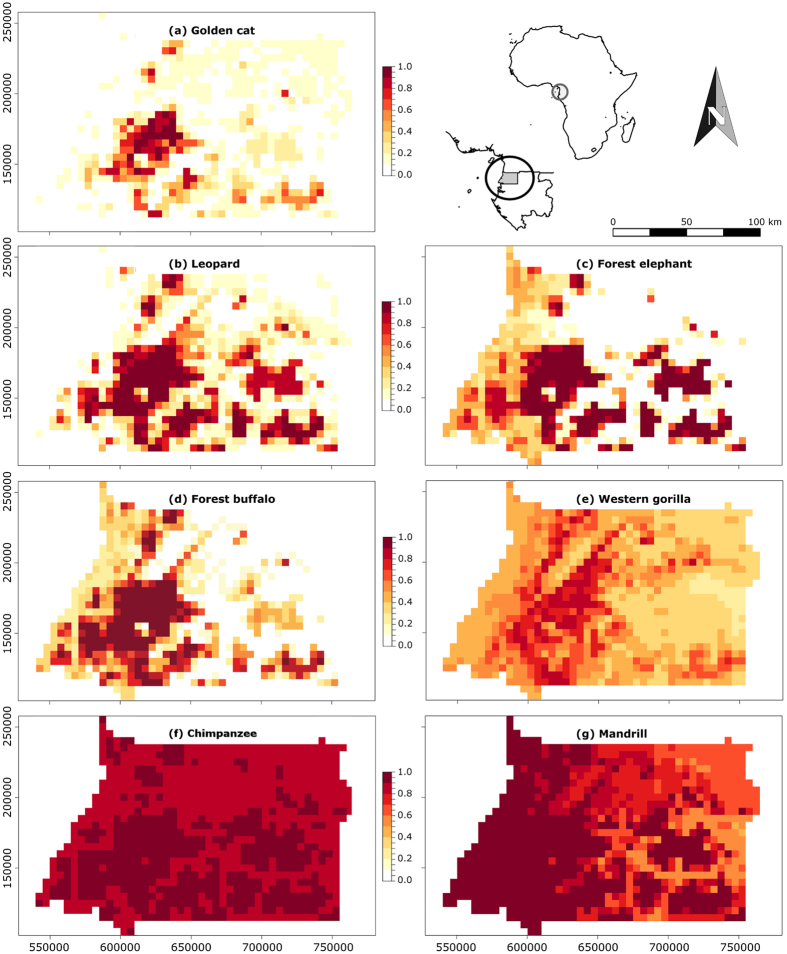
Estimated probabilities of occurrence for threatened mammals in Río Muni (Equatorial Guinea). All scales are occurrence probability per 25-km^2^ grid cell, calculated with the model averaging technique considering the most important models for each species showed in Supplementary Table S2. UTM coordinates are in zone 32N. Background maps in this figure have been generated by the authors using R version 3.2.5 (https://www.R-project.org/), and they have been incorporated in the corresponding panel using GIMP version 2.8.14 (https://www.gimp.org/). Outline maps of Africa and Equatorial Guinea has been drawn in R version 3.2.5 (https://www.R-project.org/) by using the *maps* and *mapdata* libraries (https://cran.r-project.org/web/packages/). Panels have been assembled with GIMP version 2.8.14 (https://www.gimp.org/).

Using the model averaging technique with top models for each species, we calculated average probabilities of occupancy and detection (Table 2) and developed corresponding occurrence maps for each species (Fig. 1). For most species, the average of the estimated probabilities of occupancy (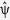) was lower than the naïve estimate (i.e. observed proportion of occupied sites). The model-averaged estimates of the probabilities of occurrence (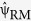) and detection (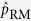) in all the cells of Río Muni region are shown in Table 2. Felids had restricted ranges (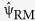 = 0.19 for golden cat and 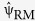 = 0.35 for leopard), especially golden cat throughout the area (Fig. 1a) and leopard in the northern half area of Río Muni (Fig. 1b). Forest elephant and forest buffalo had similar estimated occupancy probabilities (Table 2, Fig. 1c,d, respectively), although forest buffalo had a high estimated occurrence probability in the southwestern area. Among primate species, the estimated probabilities for the entire region ranged from 0.51 for gorilla, with a high distribution in the western and central areas of Río Muni (Fig. 1e), and 0.84 and 0.92 for mandrill and chimpanzee, respectively (Table 2), which both had a high distribution throughout the study area (Fig. 1f,g). The model-averaged estimates of the probability of detection were low for the two cat species and forest buffalo, but close to 1 for the rest of species (Table 2).

## Discussion

In order to study mammal occurrence, several survey designs have used camera traps^32^, faecal DNA^33^ or field surveys of easily detectable signs (dung or tracks)^34^. However, conservationists working in large and little known areas that are difficult and costly to survey, are increasingly using interviews with local knowledgeable individuals, especially hunters^15^,^17^,^18^. In light of ever increasing costs of wildlife research and conservation, inexpensive methods like this warrant special attention. In tropical forest regions the cost-effectiveness of classical survey methods (e.g. transect surveys) for the monitoring of wildlife population trends is limited^35^,^36^, and the resources available for monitoring are much smaller than in other areas^14^,^37^. In this context, there is great deal of potential for combining social surveys and occupancy analysis in large-scale biodiversity studies^4^,^6^,^17^,^19^, especially when alternative methods are limited by personnel, time, accessibility, and budget constraints^15^. Compared with another countrywide study that used standard distance line transect surveys to assess the distribution and abundance of mammals in Río Muni^13^, our hunter interview survey had the advantage of being cost-effective, requiring less personnel and field work, thus resulting in a lower budget overall. This method, based on a simple interview protocol (see Supplementary Methods for a detailed description), may have a significant potential for application to other areas in forested nations in West and Central Africa, where hunting activities are widespread and the status of medium- to large-size mammal populations is poorly known.

Nevertheless, wildlife knowledge may vary depending on the species considered. In our case, some of the study species are charismatic animals or a source of meat and therefore they are easily identified (e.g. elephants and mandrills), although others can be elusive and rare, such as forest felids. In the latter species (golden cat and leopard) this is likely to result in misidentification and so analytical approaches should be considered to improve the interpretation of biological survey data, even with modest amounts of observer experience, training, or good survey protocols^27^,^38^. Although experts are especially selected for being interviewed, misidentification can occur in field settings and their false positive estimates can be accommodated in the models. In our study, we selected knowledgeable hunters, but their answers were voluntary and could be “overestimated”. Although we selected expert hunters avoiding the most common misclassification errors in sampling methods (the effect of observer experience)^9^,^27^, hunters may be prone to record a species as present when it is in fact absent, especially when the animals are charismatic mammals or when political controversies exist^31^. The approach used in this study corroborates the findings of Miller *et al*.^31^, in which false positives were a significant component of species occurrence data collected by interview methods.

Most studies, including ours, have low rates of misclassification, although this is relevant since very small values can have a large effect on apparent occupancy^22^. Even low levels of false positive errors, constituting as little as 1% of all detections, can cause severe overestimation of site occupancy, colonization and local extinction probabilities^26^. For these reasons, we suggest that considering all forms of observation error, including false positives, provides more reliable estimates of occupancy for use in conservation and management programs^19^.

The coefficients of all covariates in the occupancy model had the same tendency (positive or negative) for the species which they influenced. Elevation had a negative relation with occurrence, indicating that lowest areas were suitable for most species. The coefficients for ruggedness, in contrast, had a positive tendency for the studied mammals, suggesting that highly variable topography provides some protection against human-induced disturbance by restricting human movements to more accessible areas. Forest areas also had a positive effect on occupancy, especially for elephants and buffalos, which need very extensive areas of forest to meet their ecological requirements. The density of villages was negatively correlated with occurrence, as expected for a landscape dominated by wildlife-dependent human populations. Similar tendencies were shown in a study that analysed these covariates in relation with the local extinction of both carnivores and herbivores in India^4^.

Top models for felids show that the density of villages was negatively correlated with occurrence, unlike terrain ruggedness, which was positively related with both species. Our results support the assumption that in a human-dominated landscape, the distribution and occurrence of felids in different habitat types are much more likely to be determined by human activities than by actual habitat preferences. The main threat for felids in Río Muni is habitat fragmentation due to the transport infrastructure^39^. As a result of human settlements along roads, the infrastructures may act as a permanent barrier to the movement of cats, so habitat corridors are needed to maintain dispersal routes between areas containing suitable habitat^7^. This approach represents major progress towards understanding the critical status of the country’s Equatoguinean cats, and a first step towards including them as a crucial component of the national plan for biodiversity conservation^11^.

In agreement with our hypothesis, the density of villages was the main covariate explaining the probability of occurrence of forest elephants, with a negative effect, while the size of forest patches had a positive effect. Forest area was also one of the main covariate explaining the probability of occurrence of forest buffalos, suggesting that habitat fragmentation could be the main threat, as Kiffner *et al*.^10^ found. The main threat to elephants in our study area is the expansion and rehabilitation of road networks, which cut off routes between local populations of forest elephants, especially between Monte Alén National Park (Río Muni) and northern Gabon^40^.

Primate populations have been declining for the last decades in Africa^41^. Gorillas are critically endangered^42^. The current geographic range of the gorilla in Río Muni, as inferred from our survey, was very similar to that described by Gonzalez-Kirchner^43^, and is mostly due to climatic variability and forest history, with a limited influence from human activity^44^. Chimpanzees showed a very high probability of occupancy across Río Muni, their greater behavioural flexibility than mandrills, enabling them to survive in human modified landscapes^45^. Gaps in chimpanzee distribution roughly match those areas with a long history of commercial agricultural activity and the most densely populated areas. Although mandrill is an elusive primate and it is difficult to study in its rain-forest habitat in equatorial Africa, its distribution in the study area is higher than that of gorilla, reflecting its less threatened status. Nevertheless, a study in Gabon showed that mandrills are threatened by hunting pressure and habitat loss^46^.

Summarizing the distribution range of mammals in Río Muni region, maps on the occurrence of the studied species (Fig. 1) showed two different macro-zones, with the southern half of the study area showing the highest occurrence. This mammal distribution can be explained by different human threats in Río Muni. Whereas the northern half of Río Muni contains higher human populations, and has been historically subjected to logging and commercial agricultural activity, the southern half is still heavily forested and sparsely populated^39^. In the northern zone the majority of large mammal populations are confined to small and isolated forest patches, and the implementation of corridors linking habitat remnants seems both unattainable and unfeasible, given the highly populated landscape matrix. The only area with remaining potential for the long-term conservation of these species is the rugged and sparsely populated tract of forest in the northwest corner of Río Muni, contiguous with Cameroon’s Campo-Ma’an National Park. There are several important reasons for pursuing some level of conservation here, given that in addition to holding leopard, golden cat, elephant and ape populations, this area is the last refuge for the hippopotamus (*Hippopotamus amphibius*) in the country^47^.

Nevertheless, given that large and relatively intact areas are required to support viable populations of our focal species, major conservation efforts could beneficially focus on the still heavily forested and sparsely populated southern half of Río Muni, and especially in its western area, where our models predict the highest probabilities of occurrence for all the studied species. This area, covered by the Monte Alén National Park, spreads south towards Monts de Cristal National Park, in neighbouring Gabon, providing opportunities for the promotion of transboundary conservation efforts^13^.

We have identified important landscape attributes and threats, which influence species occurrence and critical areas for conservation, including opportunities for future cross-boundary cooperation. Since identification of meaningful covariates is important for understanding species distribution^48^, a future step is to consider their spatial and temporal trends in order to obtain more reliable data on the distribution of species^6^. Overall, our results suggest that anthropogenic impacts (fragmentation, habitat loss and hunting) are the main factor affecting mammal occurrence, and our models defined areas with a high occurrence of mammals, represented mainly by the southern half of Río Muni. A previous study^13^, which focused on apes, elephants and global mammal species richness, identified the same areas of conservation interest in Río Muni that our models do. In agreement with Murai *et al*.^13^, we suggest that priority conservation strategies for ensuring the persistence of large mammal populations could focus on mitigating the negative effects on occurrence (e.g. through environmental law enforcement) and protect the areas identified as containing a high number of mammal species, also involving the implementation of transboundary cooperation plans^49^.

## Methods

### Study area and species

The study area covers the continental area of Equatorial Guinea (Central Africa), the 26000 km^2^ rectangular-shaped Río Muni region (Supplementary Fig. S1). Río Muni is characterized by a complex topography varying in form with distance from the Atlantic coast. The western littoral zone (less than 200 m a.s.l.) extends in a flat band 20–30 km from the coast, and is separated by the plains of the interior by the Niefang mountain range (1250 m a.s.l.) which itself runs parallel to the coast in a southwest-northeast direction. To the east of this range the hinterland peneplain (300–650 m a.s.l.) characterized by smoother gradients of elevation and studded with granite inselbergs and bisected by the main Uoro river. The climate is hot and humid throughout the year. Rainfall varies from 400 mm on the highest parts of the mountains to 1800 mm on the peneplains^37^.

Forest cover is estimated at 78%, but, given that timber was the main source of foreign exchange from the 1920s to the 1990s, virtually all the accessible areas have, at one time, been selectively logged, resulting in a mosaic matrix of secondary forest in different stages of regeneration. Despite this, significant areas of Río Muni have maintained a very high level of species richness and endemism, resulting in one of the Highest Priority Areas for conservation in Central Africa^50^. Although hunters have near complete access to wildlife (less than 10% of these forests are classified as low-access and the density of logging roads −0.09 km/km^2^– is the highest for Central Africa^51^), Río Muni contains most of the mammals typically found in the Lower Guinea Forest Block^52^. Prominent examples include the study species.

The average human population density is 19 inhabitants/km^2^, but is much lower (less than 5 inhabitants/km^2^) in areas located far from the urban centres and main public roads, along which most villages concentrate, a pattern particularly evident in the southern half of the territory. About 62% of the population relies directly on subsistence agriculture, supplemented by fishing and hunting, as the main sources of protein and regular income^37^. Although the discovery in 1992 of large-scale oil reserves has caused a shift in attention away from logging, persistent threats to wildlife such as commercial bushmeat hunting and infrastructure development (particularly road building and urban expansion) have increased considerably in the last few years^12^.

### Study design: sampling units, expert interview surveys and covariates

A large-scale interview survey was conducted between April and October 2010 to gather information on the distribution of and threats to felids and other medium- to large-size mammals in Equatorial Guinea. We collected presence/absence data derived from interviews with local hunters, which is regarded as a suitable initial step for gathering baseline information on these species. In total 138 villages and 225 experienced hunters were selected to provide a representative sample and adequate coverage of the entire region. In order to estimate occupancy, a sampling unit (site) of 5 × 5 km was defined for each hunter’s hunting grounds, encompassing a total area of 5625 km^2^ or 21.6% of the mainland territory of the country (Supplementary Fig. S1). Six hunting zones within each site were used as spatial replicates (a total of 1350 plots) to address the issue of detection probability^16^,^21^. To clearly delineate each hunter’s hunting zones we located on a georeferenced map specific areas characterised by recognisable features indicated by the interviewees. Presence records for a given species were defined as plot-level occurrences when interviewees had no doubt that the species was locally present at the time of interview. The study was approved by the Ministry of Agriculture and Forest of Equatorial Guinea and the District Government Delegates, which provided research permits to develop the fieldwork phase of the study. Interviews were carried out in accordance with the approved guidelines. All interviewees participated on a voluntary basis and gave informed consent prior to the interview. A detailed description of the interview survey is provided in Supplementary Methods.

As occupancy and detection probabilities vary according to the characteristics of each site, we selected the site-level covariates most likely to influence both detection and occurrence of large-scale mammals in Equatorial Guinea. These included landscape characteristics and human influence (Supplementary Table S1). For covariates representing landscape characteristics, we used mean site elevation, site ruggedness and extent of forest area for each 5 × 5 km cell. The density of human settlements in each site was used as a measure of human influence.

### Data analysis and model selection

We used single season occupancy models (OMs) in order to estimate functions of species occurrence and to identify factors that are associated with changes in the probability of a site being occupied (ψ). Our sampling design considered *M* = 225 sites in which we recorded the binary response *Y*_*ij*_ of species detection (*Y* = 1) or non-detection (*Y* = 0) in *J* = 6 spatial replicates (plots) within the *i*th site^2^,^5^. This hierarchical model describes the joint distribution of the observations conditional on the latent occupancy state, and the marginal distribution of the latent occupancy state variable:


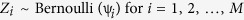



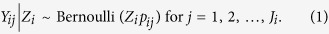


The detection probability parameter *p_ij_* accounts for imperfect observation of occupancy state, and is defined as the probability of detecting a species given that it is present. Replicate samples (*J* = 6) provide information about the detection rate separate from the occupancy rate. We modelled occupancy and detection probabilities as functions of site-level covariates using the logit link function^2^. Variables that are related to the occupancy state are modelled as


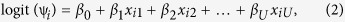


which is a function of *U* covariates associated with site *i* (*x*_*i1*_, *x*_*i2*_, …, *x*_*iU*_) and the *U* + 1 parameters that are to be estimated: an intercept or constant term (*β*_0_) and *U* regression coefficients for each covariate. Similarly, the probability of detecting species at site *i* during survey *j* could be expressed as:


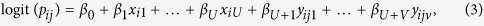


where *x*_*i*1_, …, *x*_*i*U_ denote the *U* covariates associated with site *i*, and *y*_*ij*1_, …, *y*_*ijv*_ are the *V* survey-specific covariates associated with survey *j* of a vector of site *i*.

In addition to coefficients for covariates, misclassification models also estimate a false positive rate parameter (*p*_10_), which is the probability of falsely detecting the species in an unoccupied site.

Given the large number of potential candidate models to evaluate for estimating occupancy and detection probabilities (even with a reduced number of covariates), model fitting was conducted following a two-phase approach. First, model selection was performed on the occupancy and detection parameters using conventional OMs and considering all the possible combinations of the standardized covariates, including their interactions. Maximum likelihood estimates were obtained using the *occu* function from the R package *unmarked*^53^, standardizing the covariates. Models were ranked using the difference in Akaike’s information criterion (ΔAIC) between each model and the best model (smallest AIC)^54^.

Once the best OMs were identified for each species, the second phase was to perform their corresponding misclassification models (models with false positive errors; MMs). We used the *occuFP* function from the *unmarked* package^53^, which allowed us to obtain false positive estimates in models with ψ and *p* estimates with covariates.

### Model averaging for parameter estimates and probability occurrence maps

To account for model selection uncertainty we used model-averaging^54^ of the best and alternative models (with ΔAIC values < 2.0) to estimate the average probability of occupancy and detection for each species for the whole study area, and also to draw the occurrence maps^16^ using the 961 grid cells of 25 km^2^ covering the entire Río Muni region. To perform such model averaging, we considered the model Akaike weights (*w*) given in Supplementary Table S2, which were distributed relatively evenly among the best OMs and MMs together, so that all the weights for each species add up to 1.

## Additional Information

**How to cite this article**: Martínez-Martí, C. *et al*. Integrating occurrence and detectability patterns based on interview data: a case study for threatened mammals in Equatorial Guinea. *Sci. Rep.* **6**, 33838; doi: 10.1038/srep33838 (2016).

## Supplementary Material

Supplementary Information

## Figures and Tables

**Table 1:** False positive rate parameter and untransformed coefficients of covariates estimated by the top-ranked occupancy models for threatened mammals in Río Muni (Equatorial Guinea).

Model	*p*_10_	elev	rug	for	pop	Interaction term
**Golden cat (*****Caracal aurata***)						
ψ (rug * pop + for)	0	–	0.448 (0.236)	−0.575 (0.297)	−0.990 (0.365)	rug * pop: −0.373 (0.289)
*p* (elev + pop)		0.624 (0.207)	–	–	−0.205 (0.212)	–
**Leopard (*****Panthera pardus***)						
ψ (pop + rug)	0.019	–	0.826 (0.222)	–	−1.578 (0.444)	–
*p* (pop * elev + for)		0.943 (0.251)	–	0.431 (0.175)	−1.565 (0.363)	pop * elev: 2.007 (0.422)
**Forest elephant (*****Loxodonta cyclotis***)						
ψ (pop * elev + for)	0.022	−0.641 (0.208)	–	0.589 (0.321)	−1.968 (0.431)	pop * elev: −0.757 (0.313)
*p* (for * pop)		–	–	0.672 (0.528)	−0.155 (0.303)	for * pop: 0.139 (0.699)
**Forest buffalo (*****Syncerus caffer***)						
ψ (for + elev + rug + pop)	0	−1.513 (0.265)	1.282 (0.275)	0.051 (0.391)	−1.471 (0.423)	–
*p* ((for + rug + elev) * pop)		−1.398 (0.275)	0.858 (0.242)	−0.047 (0.304)	−1.184 (0.385)	for * pop: −2.414 (0.399) rug * pop: 0.724 (0.345) elev * pop: −1.786 (0.402)
**Western gorilla (*****Gorilla gorilla***)						
ψ (rug + elev)	0.020	−0.364 (0.155)	0.730 (0.174)	–	–	–
*p* (pop * rug + elev)		−0.216 (0.134)	0.053 (0.113)	–	−0.217 (0.125)	pop * rug: −0.487 (0.107)
**Chimpanzee (*****Pan troglodytes***)						
ψ (pop)	0.056	–	–	–	−1.400 (0.253)	–
*p* (pop * rug)		–	0.063 (0.121)	–	−0.438 (0.110)	pop * rug: −0.283 (0.098)
**Mandrill (*****Mandrillus sphinx***)						
ψ (pop + elev + rug)	0.005	−0.915 (0.349)	0.687 (0.312)	–	−1.730 (0.276)	–
*p* (pop * elev + rug + for)		−0.073 (0.114)	0.250 (0.122)	0.189 (0.133)	−0.757 (0.144)	pop * elev: −0.379 (0.139)

Note that the false positive rate parameter (*p*_10_) is null in non-misclassification models. Asterisk indicates an interaction model. Standard errors are given in parentheses. Covariates: elevation (elev), ruggedness (rug), human population (pop), forest area (for).

**Table 2:** Summary of the occupancy and detection probability estimates for threatened mammals in Río Muni (Equatorial Guinea).

Species	IUCN red list category^a^	Population trend^a^	x	Naïve estimate of ψ	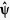	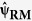	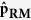
Golden cat	Vulnerable	Decreasing	36	0.16	0.17	0.19	0.49
Leopard	Near threatened	Decreasing	101	0.45	0.28	0.35	0.47
Forest elephant	Not evaluated	Decreasing^b^	99	0.44	0.40	0.34	0.87
Forest buffalo	Least concern	Decreasing	79	0.35	0.36	0.33	0.51
Western gorilla	Critically endangered	Decreasing	126	0.56	0.51	0.51	0.95
Chimpanzee	Endangered	Decreasing	207	0.92	0.90	0.92	0.84
Mandrill	Vulnerable	Unknown	189	0.84	0.84	0.84	0.92

The number of cells in which a species was detected = *x* and the number of plausible cells within which a species might occur is 225. The naïve estimate of occupancy for surveyed cells is ψ = *x*/*225*. 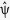 is the model-averaged estimate of the probability of occupancy for sites, computed as the sum of occupancy probabilities for all surveyed cells divided by the number of sites. 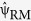 and 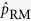 are the model-averaged estimates of the probabilities of occupancy and detection, respectively, in all 25 km^2^ cells in Río Muni.

^a^Status and population trend in the world obtained from IUCN^55^.

^b^Population trend for the forest elephant from Maisels *et al*.^56^.
