# Supplementary material for: Integrating occurrence and detectability patterns based on interview data: a case study for threatened mammals in Equatorial Guinea
Source: Sci Rep. 2016 Sep 26;6:33838. doi: 10.1038/srep33838 (PMC5036030; doi:10.1038/srep33838)
Supplement: Supplementary Information [file srep33838-s1.pdf]

Supplementary Information

Title: Integrating occurrence and detectability patterns based on interview data: a case study for threatened mammals in Equatorial Guinea

Authors: Chele Martínez-Martí[1], María V. Jiménez-Franco[1], J. Andrew Royle[2], José A. Palazón[1] & José F. Calvo[1]

[1]Departamento de Ecología e Hidrología, Facultad de Biología, Universidad de Murcia, Campus de Espinardo, 30100 Murcia, Spain.

[2]USGS Patuxent Wildlife Research Center, 12100, Beech Forest Road, Laurel, MD 20708, USA.

Supplementary Methods. Description of the interview survey.

In all villages surveyed, the first activity involved meeting the village chief to introduce ourselves and the study, plan activities and to consult him on the selection of hunters to be interviewed. Our interview requirements for a hunter were that he should still hunt, should have hunted regularly in the same area, and should be considered an expert by his peer group in the village. This resulted in the selection of people with intimate knowledge of the area and its wildlife and who were therefore able to list which species can be found and in which specific sites.

To clearly delineate each hunter's hunting zones we located on a georeferenced map specific areas characterised by recognisable features indicated by the interviewees. The respondents were asked to comment only on species occurring within these recently delineated zones. These areas were invariably small allowing for the repeatable recall of estimated presence/absence from the hunter's memory.

During the interviews a species list was provided in Fang, the local dialect, alongside photographs of each animal. To assess the frequency of type II errors, our photographs included several species known to be entirely absent from the study site although morphologically similar to the target species. In all trials, interviewees never falsely identified a species known to exclusively occur outside of the region as present within their forest patches. This gave us confidence that information provided on species identity was reliable. Presence records for a given species were defined as plot-level occurrences when interviewees had no doubt as to whether the species was locally present at the time of interview and whether the species was thought to be a full-time resident within the patch.

Spatial accuracy of hunter reporting was assessed by comparing distances and bearings reported from interviewees to neighbouring villages against actual distances to the same

villages previously measured from maps. In most cases we found a high degree of accuracy between perceived and map distances and bearings.

Interviewees were relaxed and open on the subject during all our conversations. As enforcement of official restrictions on bushmeat hunting and trade is non-existent in the country, regulations are seldom observed and thus interviewees talked openly about their hunting experiences providing us with comprehensive and unbiased responses. Reliability of hunter information on presence-absence of wildlife in a site was assessed by conducting additional interviews in neighbouring villages where hunters were known to be active in the same site.

The study was approved by the Ministry of Agriculture and Forest of Equatorial Guinea and the District Government Delegates, which provided research permits to develop the fieldwork phase of the study. Interviews were carried out in accordance with the approved guidelines. All interviewees participated on a voluntary basis and gave informed consent prior to the interview.

Supplementary Figure S1. Map of the study area, Rio Muni region, the continental area of Equatorial Guinea in Central Africa, showing the 225 sites (5 x 5 km) surveyed in this study. The main map in this figure and the outline maps of Africa and Equatorial Guinea have been drawn in R version 3.2.5 (https://www.R-project.org/) by using the *maps* and *mapdata* libraries (https://cran.r-project.org/web/packages/). The figure has been assembled with inkscape version 0.91 r13725 (https://inkscape.org).

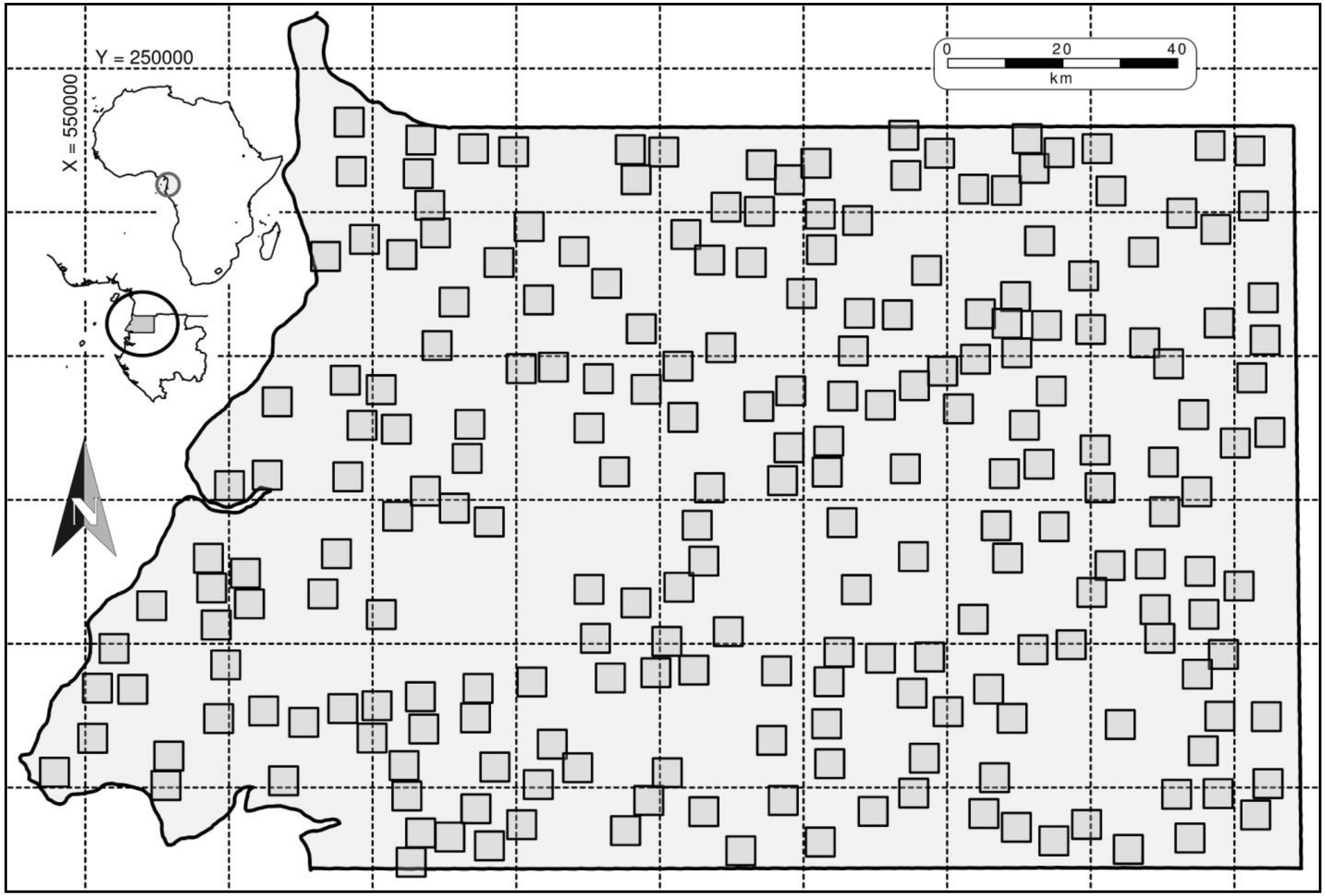

Supplementary Tables

Supplementary Table S1. Predicted relationships between covariates related to landscape characteristic (elevation, ruggedness, forest area) and human influence (population density) with occupancy of threatened mammals in Equatorial Guinea. The strengths and direction of predicted trends are indicated with positive and negative signs. Zeros indicate that no relationship between occupancy and the predictor is expected. C = golden cat (*Caracal aurata*), L = leopard (*Panthera pardus*), E = forest elephant (*Loxodonta cyclotis*), B = forest buffalo (*Syncerus caffer*), G = western gorilla (*Gorilla gorilla*), Ch = chimpanzee (*Pan troglodytes*), M = mandrill (*Mandrillus sphinx*). [a]Source: Instituto Nacional de Desarrollo Forestal y Gestión del Sistema de Áreas Protegidas (INDEFOR-AP) of Equatorial Guinea. [b]Source: Central Africa Regional Program for the Environment (http://carpe.umd.edu; accessed 17 Nov 2011).

**Covariate**	**Description**	**Influence**	**Predictions for occupancy**
Elevation[a]	Average elevation in a sample unit of 25 km^2. Original resolution: 30 m.	Elevation influences the distribution of wildlife through different types of vegetation that are associated with different altitudes.	C (0), L (0)<br>E (0), B (– –)<br>G (0), Ch (0), M (0)
Ruggedness[a]	Average slope in a sample unit of 25 km^2. Original	Ruggedness influences the distribution of wildlife species by restricting human activities to more	C (+ + +), L (+ +)<br>E (0), B (+)

	resolution: 30 m.	accessible.	G (+ + +), Ch (0), M (0)
Forest area[b]	Extent of forest in a sample unit of 25 km^2. Original resolution: 30 m.	Large mammal native to Central Africa require very extensive areas of forest, either because they have inherently large habitat requirements or because it enables them to move away from human disturbance and chronically hunted areas.	C (+ + +), L(+ +)<br>E (+ + +), B (+ + +)<br>G (0), Ch (0), M (0)
Human population density[a]	Number of human settlements (human villages) in a sample unit of 25 km^2.	Area used by large mammals in Central Africa is best explained by disturbance from human settlements.	C (– – –), L (– –)<br>E (– –), B (– – –)<br>G (0), Ch (–), M (–)

Supplementary Table S2. Summary of model selection for estimation of occupancy (ψ) and detection probability (*p*) in threatened mammals in Equatorial Guinea. Only the six best single-season occupancy models (conventional models) for each species are shown, together with their corresponding misclassification models (models with false positive errors). AIC is the Akaike information criterion, *w* is the Akaike weight, *n* is the number of parameters in the model, ΔAIC is the difference in AIC values between each model and the best model (lowest AIC), and p_{10} is the false positive rate parameter in misclassification models. Covariates: elevation (elev), ruggedness (rug), human population density (pop), forest area (for). The top ranked models for each species (with ΔAIC < 2.0) are shown with bold AIC. Note that p_{10} is null in conventional models, and all the Akaike weights for each species add up to 1.0 precisely.

	**Conventional models**				**Misclassification models**				
	**AIC**	***w***	***n***	**ΔAIC**	**AIC**	p_{10}	***w***	***n***	**ΔAIC**
**Golden cat (*Caracal aurata*)**									
ψ (rug * pop + forest), *p* (elev + pop)	**473.00**	0.32	8	0.00	476.83	0.001	0.05	9	3.83
ψ (rug * pop), *p* (elev)	**473.73**	0.22	6	0.73	476.09	0.002	0.07	7	3.10
ψ (rug * pop), *p* (elev + pop)	**474.72**	0.14	7	1.72	478.00	0.002	0.03	8	5.00
ψ (rug * pop + forest), *p* (elev + pop + forest)	**474.92**	0.12	9	1.92	478.73	0.002	0.02	10	5.73
ψ (rug), *p* (elev)	478.25	0.02	4	5.25	482.05	0.001	0.00	7	9.06
ψ (rug * pop), *p* (pop)	483.42	0.00	6	10.42	481.01	0.005	0.01	5	8.01

**Leopard (*Panthera pardus*)**									
ψ (pop + rug), *p* (pop * elev + forest + rug)	987.28	0.00	9	26.36	**962.90**	0.019	0.25	10	1.99
ψ (pop + rug), *p* (pop * elev + forest)	987.56	0.00	8	26.65	**960.91**	0.019	0.66	9	0.00
ψ (pop + rug), *p* (pop* elev)	994.90	0.00	7	33.99	964.86	0.021	0.09	8	3.94
ψ (pop + rug + forest), *p* (pop+ elev +forest + rug)	995.82	0.00	9	34.91	974.60	0.030	0.00	10	13.69
ψ (pop + rug), *p* (pop + forest)	999.62	0.00	6	38.71	976.26	0.022	0.00	7	15.35
ψ (pop + forest), *p* (pop + forest +rug)	1016.69	0.00	7	55.77	992.25	0.022	0.00	8	31.34
**Forest elephant (*Loxodonta cyclotis*)**									
ψ (pop * elev + forest), *p* (forest * pop)	708.62	0.00	9	32.41	**676.21**	0.022	0.53	10	0.00
ψ (pop * elev), *p* (forest * pop)	710.42	0.00	8	34.21	**677.82**	0.022	0.24	9	1.61
ψ (pop * elev + forest), *p* (forest * pop + elev + rug)	710.66	0.00	11	34.45	680.17	0.022	0.07	12	3.96
ψ (pop * elev), *p* (forest * pop + elev)	712.34	0.00	9	36.13	679.81	0.022	0.09	10	3.60
ψ (pop + elev + forest), *p* (forest * pop)	713.32	0.00	8	37.11	680.06	0.022	0.07	9	3.85
ψ (pop * forest), *p* (forest * pop + elev)	721.42	0.00	9	45.21	686.87	0.022	0.00	10	10.66
**Forest buffalo (*Syncerus caffer*)**									
ψ (forest + elev + rug + pop), *p* ((forest + rug + elev) * pop)	**702.38**	0.65	13	0.00	**703.59**	0.011	0.35	13	1.22

ψ (forest + elev + rug + pop), *p* (forest + rug + elev + pop)	741.91	0.00	10	39.53	732.84	0.024	0.00	10	30.47
ψ (forest + elev + rug), *p* (forest + rug + elev)	759.14	0.00	8	56.76	752.72	0.009	0.00	8	50.34
ψ ((forest + elev) * rug), *p* ((forest + rug) * elev)	761.46	0.00	12	59.08	757.13	0.008	0.00	12	54.75
ψ (forest + elev + pop), *p* (forest + rug + pop)	775.61	0.00	8	73.23	762.21	0.014	0.00	8	59.84
ψ (forest + elev), *p* (forest + rug)	790.00	0.00	6	87.63	776.94	0.023	0.00	6	74.56
**Western gorilla (*Gorilla gorilla*)**									
ψ (rug + elev), *p* (pop * rug + elev)	1121.24	0.00	9	39.03	**1082.21**	0.020	0.52	9	0.00
ψ (rug + elev), *p* (pop * rug + forest)	1123.63	0.00	11	41.42	1084.43	0.021	0.17	9	2.23
ψ (rug + elev + pop + forest), *p* (pop * rug + elev)	1124.32	0.00	9	42.11	1086.02	0.020	0.08	11	3.81
ψ (rug * elev), *p* (pop * rug + elev + forest)	1125.07	0.00	11	42.87	1084.22	0.019	0.19	11	2.01
ψ (rug), *p* (pop * rug)	1127.43	0.00	7	45.23	1087.71	0.022	0.04	7	5.51
ψ (rug + elev + forest), *p* (pop + rug + elev)	1138.43	0.00	9	56.23	1103.96	0.034	0.00	9	21.76
**Chimpanzee (*Pan troglodytes*)**									
ψ (pop), *p* (pop * elev + forest)	862.37	0.00	7	31.06	837.77	0.054	0.03	8	6.46
ψ (pop + forest), *p* (pop * elev + forest)	862.66	0.00	8	31.35	836.16	0.056	0.06	9	4.84
ψ (pop), *p* (pop * rug)	862.93	0.00	9	31.61	**831.31**	0.056	0.71	7	0.00

ψ (pop + rug), *p* (pop * elev)	863.55	0.00	8	32.24	834.34	0.057	0.16	8	3.03
ψ (pop), *p* (pop * elev + forest + rug)	864.15	0.00	8	32.84	839.67	0.054	0.01	9	8.36
ψ (pop), *p* (pop * elev + rug)	864.19	0.00	9	32.88	837.85	0.056	0.03	8	6.54
**Mandrill (*Mandrillus sphinx*)**									
ψ (pop + elev + rug), *p* (pop * elev + rug)	**880.84**	0.16	9	0.69	**880.15**	0.005	0.21	10	0.00
ψ (pop + elev + rug), *p* (pop * elev + rug + forest)	**881.14**	0.13	10	0.99	**880.15**	0.005	0.21	11	0.00
ψ (pop + elev + rug + forest), *p* (pop * elev + rug + forest)	882.72	0.06	11	2.57	**881.84**	0.005	0.09	12	1.69
ψ (pop + elev), *p* (pop * elev + rug)	883.06	0.05	8	2.91	883.68	0.005	0.04	9	3.53
ψ (pop), *p* (pop * elev + rug)	885.31	0.02	7	5.16	884.71	0.079	0.02	8	4.57
ψ (pop + elev), *p* (pop * elev + forest)	888.69	0.00	8	8.54	886.21	0.005	0.01	9	6.06
